# Quantitative Whole‐Body Muscle MRI in Adults With Spinal Muscular Atrophy–A Sensitive Tool for Long‐Time Evaluation of Disease Progression

**DOI:** 10.1111/ene.70579

**Published:** 2026-03-23

**Authors:** Alexander Mensch, Benjamin Troppa, Ilka Schneider, Caroline Deborah Stapf, Anna Katharina Koelsch, Thomas Kendzierski, David Strube, Sebastian Plutz, Max Obenauf, Karl‐Stefan Delank, Torsten Kraya, Markus Otto, Dietrich Stoevesandt, Steffen Naegel

**Affiliations:** ^1^ Department of Neurology University Medicine Halle Halle (Saale) Saxony‐Anhalt Germany; ^2^ Heimer Institute for Muscle Research Heimer Institute for Muscle Research Bochum North Rhine‐Westphalia Germany; ^3^ Department of Radiology University Medicine Halle Halle (Saale) Saxony‐Anhalt Germany; ^4^ Department of Neurology St. Georg Hospital Leipzig Leipzig Saxony Germany; ^5^ Department of Orthopedic and Trauma Surgery University Medicine Halle Halle (Saale) Saxony‐Anhalt Germany; ^6^ Department of Neurology Alfried Krupp Krankenhaus Essen Essen North Rhine‐Westphalia Germany

**Keywords:** imaging biomarker, muscular fat fraction, nusinersen, qMRI, SMA

## Abstract

**Background:**

Spinal muscular atrophy (SMA) is a neuromuscular disorder characterized by progressive muscle weakness due to SMN protein deficiency. While effective therapies exist, their impact on slowly progressive adult SMA patients remains unclear. Reliable biomarkers for monitoring disease progression and treatment response are urgently needed. This pilot study evaluated the utility of longitudinal quantitative muscle MRI (qMRI) to monitor disease progression in adult SMA patients treated with nusinersen over an extended period.

**Methods:**

Nine adult patients with genetically confirmed 5q‐SMA underwent whole‐body muscle MRI and clinical assessment, including the Hammersmith Functional Motor Scale‐Expanded (HFMS‐EXP), Revised Upper Limb Module (RULM), and 6 min walk test (6MWT). Muscular fat fraction (mFF) was quantified in 20 muscles over a median follow‐up of 54 months.

**Results:**

Baseline mFF correlated strongly with clinical measures (HFMS‐EXP: *r* = −0.90, *p* = 0.001; 6MWT: *r* = −0.96, *p* < 0.001), but not with age at onset or age at MRI. Over the observation period, a significant increase in mFF was detected (averaged annual increase of all studied muscles: 0.47%, *p* = 0.011), accentuated in the lower leg muscles. In contrast, clinical measures showed no consistent change. Consequently, no significant correlations were found between changes in mFF and clinical scores.

**Conclusions:**

This study provides the longest reported longitudinal qMRI assessment in adult SMA patients treated with nusinersen, demonstrating that mFF progressively increases despite stable clinical scores. The results suggest that qMRI may be a sensitive and objective biomarker for detecting subtle disease progression in adult SMA, potentially surpassing clinical measures.

## Graphical abstract

Quantitative muscle MRI is emerging as an objective biomarker for monitoring therapy in neuromuscular disorders, yet long‐term trajectories in slowly progressive adult spinal muscular atrophy (SMA) remain unclear. In this study, quantitative MRI parameters in nusinersen‐treated adults were longitudinally evaluated and compared with established clinical outcome measures. MRI detected progression with higher sensitivity than conventional measures, supporting its use as an objective addition to the SMA monitoring toolbox and motivating validation in larger cohorts.

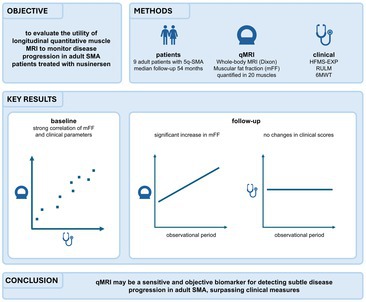

## 1 Introduction

Spinal muscular atrophy (SMA) is a rare genetic neuromuscular disorder characterized by progressive muscle weakness and atrophy due to the degeneration of lower motor neurons [1]. The underlying cause is a bi‐allelic loss of function in the *SMN1* gene, resulting in a deficiency of survival motor neuron (SMN) protein, which is crucial for the development and function of motor neurons [2, 3]. In recent years, therapeutic options for patients with 5q‐spinal muscular atrophy (5q‐SMA) have progressively expanded, culminating in the approval of three causally effective treatments: nusinersen, risdiplam, and onasemnogene abeparvovec [1]. While the latter is exclusively available for young children with early‐onset SMA, nusinersen and risdiplam have been approved for the treatment of 5q‐SMA, irrespective of the age of onset.

Although the efficacy of these therapies in symptomatic pediatric patients with 5q‐SMA is well established, their therapeutic benefits in (slowly progressive) adult patients remain insufficiently investigated given the small number of these patients in the phase III clinical trials [4, 5, 6, 7]. Although phase IV studies generally suggest a potential benefit for this patient group, the available data remain partly inconclusive and conflicting, with several questions persisting [8, 9, 10, 11, 12, 13, 14, 15, 16]. A key challenge is the slow progression of juvenile‐ or adult‐onset 5q‐SMA, which complicates the reliable assessment of treatment response. In this context, concerns have been raised regarding the sensitivity of currently employed clinical outcome measures in detecting subtle therapeutic effects on disease progression in late‐onset 5q‐SMA [17, 18]. This is especially apparent in the very advanced as well as the mildly affected patients, where floor or ceiling effects have been discussed, respectively [19]. Hence, there is an urgent need for reliable biomarkers capable of adequately monitoring disease progression over time.

Quantitative magnetic resonance imaging (MRI) of the skeletal muscle offers a non‐invasive and objective approach for the longitudinal assessment of intramuscular alterations and thus may serve as an alternative biomarker in SMA. Recent studies have demonstrated a robust correlation between various quantitative MRI parameters, disease duration, and clinical indicators of disease severity in adult SMA patients [20, 21]. Furthermore, there is evidence suggesting a predictive role of quantitative muscle MRI measurements in assessing treatment response in children [22, 23]. Accordingly, pilot studies in treated pediatric patients have shown increasing muscular fat fractions over time, supporting the potential of quantitative muscle MRI as a promising diagnostic and prognostic imaging biomarker [21]. However, longitudinal studies in adults have shown inconsistent results, particularly due to the relatively short follow‐up periods (6–12 months) employed in these investigations [20, 24, 25, 26].

In this regard, the present pilot study aimed to examine the long‐term progression of SMA in patients undergoing treatment with nusinersen using longitudinal quantitative MRI analyses.

## 2 Method

### 2.1 Patient Selection

This retrospective study included all patients with genetically confirmed 5q‐SMA treated with nusinersen (12 mg) at the Neuromuscular Center Halle who underwent whole‐body muscle MRI as part of the internal standard of care. All patients had received a baseline MRI prior to treatment initiation, and nusinersen therapy was continued throughout the entire observational period. To ensure detectable changes in muscle composition, the minimum observational period was defined as 26 months (i.e., two years after completion of the loading phase), as anticipated from natural history studies [26]. The cutoff date for included measurements was December 2023, resulting in individual observational periods ranging from 26 to 65 months. Of the 13 patients treated with nusinersen, 9 met the inclusion criteria. Two patients could not be adequately positioned in the MRI scanner due to contractures and therefore did not tolerate imaging; one patient discontinued treatment, and one had a too short observational period. Patients treated with risdiplam (*n* = 8) were excluded because of their insufficient observational periods. All MRI scans and clinical data were anonymized prior to analysis.

### 2.2 Clinical Data

Clinical data were obtained by careful review of patient records. Parameters collected and analyzed included age at MRI, age at symptom onset, sex, Hammersmith Functional Motor Scale‐Expanded (HFMS‐EXP/HFMS+), Revised Upper Limb Module (RULM) for SMA, 6‐min walk test (6MWT), and creatine kinase in serum (CK). The 6MWT was assessed only in ambulatory patients.

### 2.3 MRI Acquisition

Whole‐body MR‐imaging was performed using a 3 T MRI (Skyra; Siemens, Erlangen, Germany) with two flexible 18‐channel transmit/receive surface body coils for neck, thorax, arms, abdomen, and pelvis, and a 36‐channel angiography coil for the legs. All patients were scanned in a supine position. Imaging included a T1‐weighted Dixon Turbo Spin Echo sequence (TSE) in axial planes with 8 mm slice thickness. No sedation or contrast agent was used.

### 2.4 Quantitative MRI‐Evaluation

For all patients, a predefined set of muscles was analyzed. Muscles of the thigh and lower leg that were sufficiently large to allow for reliable measurement were included. In addition, selected muscles of the shoulder and trunk were incorporated, based on previous studies demonstrating their measurement reliability. In total, 20 muscles were assessed. The absolute fat content of these muscles was quantified using the method described by Dixon [27]. Regions of interest were determined by a radiologist with experience in neuromuscular imaging (BT) and confirmed by an expert in the field (DS). Both evaluators were blinded to both the patient data and the time point of the scan. Predefined anatomical landmarks served as references for the identification of the regions of interest. To minimize the influence of artifacts, three adjacent slices were measured, and the mean value across these slices was used for further analysis. The muscular fat fraction (mFF) was determined for each individual muscle as well as for aggregated groups as averaged value: all muscles (ALL), the upper leg (UL), and the lower leg (LL) (for detailed muscle grouping, see Table S1).

### 2.5 Statistical Analysis

Statistical analyses were performed using IBM SPSS Statistics, version 25 (IBM, Armonk, NY, USA), and JASP (JASP Team, 2023, version 0.17.1). Due to the small group sizes, normal distribution could not be assumed based on the central limit theorem; therefore, nonparametric tests were used for all statistical analyses. Longitudinal paired testing was conducted using the Wilcoxon signed‐rank test. One‐sided tests were applied where appropriate (e.g., when an increase in muscle fat fraction [mFF] at follow‐up was expected). Longitudinal MRI data were analyzed using a three‐step hierarchical approach, in which each subsequent level was only analyzed if the preceding level showed statistically significant results. At the first level, the average mean mFF of all muscles (ALL) was tested. The second level involved analysis of the averaged upper leg (UL) and lower leg (LL) mFF. The third level comprised testing of individual muscles. A few muscles (deltoid, biceps brachii, rectus abdominis, gluteus maximus) were not grouped and were therefore analyzed even without a significant result at level two. Spearman's rank correlation test was used to assess correlations. As this was a pilot study with a small sample size, no correction for multiple testing was applied.

### 2.6 Protocol Approvals and Patient Consents

The study was approved by the institutional research committee of Martin Luther University Halle‐Wittenberg (approval number 2020–215). Due to the retrospective nature of the study, informed consent was not required.

### 2.7 Declaration of AI Technologies

During the preparation of this work, the authors used ChatGPT from OpenAI to improve language and readability of parts of the text. After using this tool, the authors reviewed and edited the content as needed and took full responsibility for the content of the publication.

## 3 Results

### 3.1 Patients Characteristics and Disease Severity

Baseline characteristics of the cohort, including disease history and clinical findings, are summarized in Table 1A,B. A total of 9 patients (2 women, 7 men; median age 50 years [range: 31–66 years]) with 5q‐associated spinal muscular atrophy (5q‐SMA) were included (1 with SMA type II, 6 with type III, and 2 with type IV). The median disease duration at baseline was 26 years (range: 18–49 years), and the median observation period was 54 months (range: 26–65 months). Most patients exhibited moderate clinical impairment at baseline, reflected by relatively high median values in the clinical assessments. However, there was notable heterogeneity, as indicated by the wide range of the included scores (e.g., median HFMS‐EXP: 48; range: 2–57; see Table 1B for details). At study onset, the majority of patients (7/9) were ambulatory. During the observational period, one additional patient lost the ability to walk independently, resulting in one‐third of the cohort being non‐ambulatory at the final time point.

**TABLE 1:** Demographic and clinical data.

	Baseline median (average) [min‐max]	Follow up median (average) [min‐max]	Annual difference median (average) [min‐max]	*p*
A‐Demographics				
Age (years)	50 (48.2) [31–66]	54 (52.6) [36–71]	1	NA
Age at disease onset (years)	13 (17.1) [1.5–43]		NA	NA
Disease Duration (years)	26 (31.2) [18–49]	29.4 (35.6) [23–53]	1	NA
B‐Clinical parameter				
HFMS	38 (32.1) [2–40]	38 (33.2) [2–40]	0.21 (0.35) [−0.18–1.88]	0.967
HFMS‐EXP	48 (40.6) [2–57]	43 (40.3) [2–57]	0 (0.11) [−1.29–2.35]	0.466
RULM	37 (31.9) [12–37]	30 (30.2) [9–37]	0 (−0.2) [−1.29–1.41]	0.101
6MWT (m) ↯	365 (313.4) [67–465]	356 (336.0) [100–507]	−0.70 (−2.87) [−21.89–10.38]	0.376
Ambulation	7/9	6/9	NA	NA
CK (μkat/l)	5.1 (8.9) [1.1–24.8]	4.4 (6.0) [0.4–15.4]	−0.18 (−0.66) [−2.51–0.38]	0.027*

*Note:* Demographic (A) and clinical parameter (B) at Baseline, Follow‐Up and annual change. ↯ = one patient was excluded as he lost ambulation. *p* Values for one sided Wilcoxon‐Test comparing baseline and follow‐up where applicable. *statistically significant at *p* < 0.05.

Abbreviations: CK, creatine kinase; 6MWT, 6 min walking test; HFMS, hammersmith functional motor scale; HFMS‐EXP, hfms–expanded; RULM, revised upper limb module for spinal muscular atrophy.

### 3.2 Baseline Relationship of mFF With Clinical Impairment and Disease History

**FIGURE 1:**
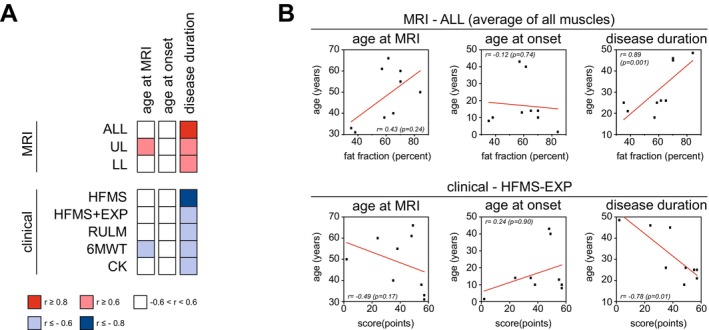
Baseline relationships between muscle fat fraction (mFF), clinical parameters, and disease history. (A) Correlation matrix showing baseline associations between key disease history variables (age at MRI, age at disease onset, disease duration), average mFF across all studied muscles (ALL), mFF of the upper legs (UL) and lower legs (LL), and clinical parameters (HFMS‐EXT, RULM, 6MWT, CK). (B) Example scatterplots illustrating the correlation between disease history variables and mFF(ALL) or clinical impairment, here shown using the Hammersmith Functional Motor Scale—Expanded (HFMS‐EXT).

At baseline, the average mFF of all studied muscles (mFF(ALL)) was neither associated with the age at disease onset (*r* = −0.13, *p* = 0.24) nor with the age at MRI (*r* = 0.43, *p* = 0.24). The same applied to clinical severity measures (HFMS(+EXP), RULM, 6MWT, CK) that did not show any relevant correlations to age at symptom onset or age at MRI. Significant correlations were observed between symptom duration and both mFF (positive correlation) and clinical scores (negative correlation) (Figure 1; Figure S1; Table S2).

**FIGURE 2:**
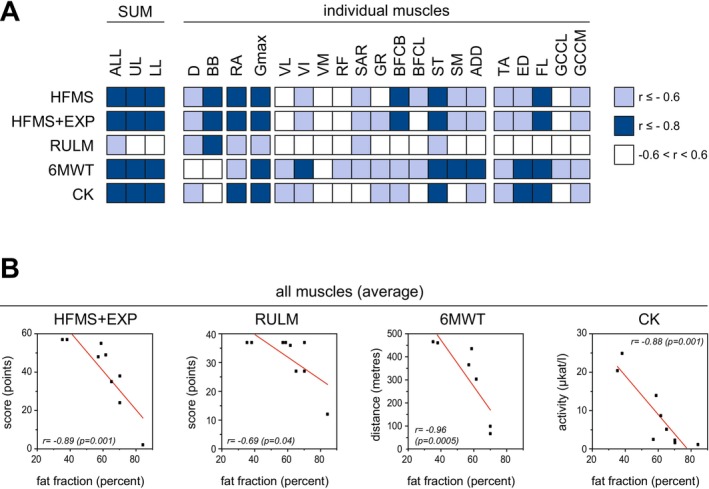
Relationships between quantitative MRI parameters and markers of clinical severity. (A) Correlation matrix depicting baseline associations between mean muscle fat fraction (mFF) across all examined muscles (ALL), the upper legs (UL), and lower legs (LL), and clinical parameters: Hammersmith Functional Motor Scale–Expanded (HFMS+EXP), Revised Upper Limb Module (RULM), 6‐Minute Walk Test (6MWT), and serum creatine kinase levels (CK). (B) Representative scatterplots illustrating the correlations between mFF averaged in all muscles (ALL) and the corresponding clinical parameters (HFMS+EXP, RULM, 6MWT, CK). Sample scatterplots for UL, LL and individual muscles can be found in Figure S2.

Regarding the reflection of clinical impairment by MRI, MRI measures showed strong correlations with clinical impairment (Figure 2A,B; Table S2A). The average muscle fat fraction across all studied muscles (mFF(ALL)) demonstrated good to excellent correlations with HFMS‐EXP (*r* = −0.92, *p* < 0.001), 6MWT (*r* = −0.96, *p* < 0.001), and CK levels (*r* = −0.88, *p* = 0.002), and a moderate correlation with RULM (*r* = −0.73, *p* = 0.02). Similar results were observed for the average mFF of the thigh and lower leg muscles, except for RULM, which showed weaker associations in these regions (Figure 2A; Figure S2; Table S2A *)*. Analysis at the level of individual muscles revealed a heterogeneous pattern. HFMS(+EXP) and 6MWT scores correlated well with several thigh muscles–particularly the hamstrings–and with muscles of the lower leg. In contrast, RULM scores did not show meaningful correlations with lower limb muscles. Instead, moderate to good correlations were observed between RULM scores and muscles of the arm, shoulder, and trunk (Figure 2A; Figure S2; Table S2‐B).

### 3.3 Longitudinal Analysis of qMRI, Clinical and Laboratory Parameters

**FIGURE 3:**
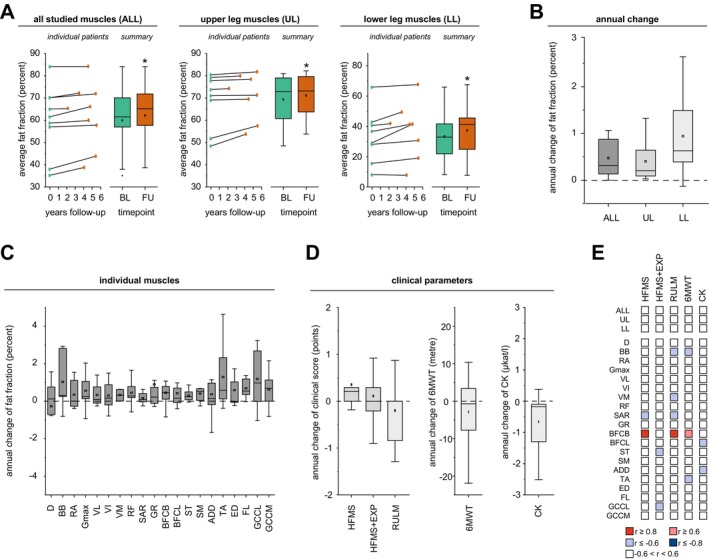
Longitudinal analysis of quantitative MRI (qMRI), clinical, and laboratory parameters. (A) Absolute changes in muscle fat fraction (mFF) across all examined muscles (ALL), as well as in muscles of the upper legs (UL) and lower legs (LL), over the observational period. Individual patient trajectories are shown with dots and lines, while box‐and‐whisker plots summarize group‐level data at baseline (BL) and last follow‐up (FU). (B) Annualized changes in mFF for ALL, UL, and LL. (C) Annualized mFF change at the level of individual muscles. (D) Annualized changes in clinical parameters: Hammersmith Functional Motor Scale (HFMS), Hammersmith Functional Motor Scale Expanded (HFMS+EXP), Revised Upper Limb Module (RULM), 6‐Minute Walk Test (6MWT), and serum creatine kinase (CK) levels. (E) Correlation matrix showing associations between the annualized change in mFF (ALL, UL, LL, and individual muscles) and changes in clinical parameters (HFMS, HFMS+EXP, RULM, 6MWT, CK).

The average mean fat fraction (mFF) of all analyzed muscles increased significantly over the observation period (average absolute change 2.18%, range: 0.009–5.79%; average annual change 0.47%, range: 0.002–1.07%; *p* = 0.011) (Figure 3A,B). Accordingly, a consistent–though heterogeneous–increase in mFF was observed in both the thighs and lower legs. Notably, the most pronounced changes occurred in the lower leg muscles (average absolute change: 4.05%, range: −0.52–12.56%; average annual change: 0.94%, range: −0.13–2.64%; *p* = 0.011), while more moderate changes were seen in the thighs (average absolute change: 1.81%, range: 0.16–5.68%; average annual change: 0.40%, range: 0.03–1.32%; *p* = 0.017).

At the level of individual muscles, similar patterns were observed. Annual increases in fat fraction were most prominent in the lower leg muscles, whereas only moderate changes were detected in the thigh muscles (Figure 3C; Figure S3). A numerical increase in the mFF of individual muscles was also noted. However, these changes were mostly non‐significant, even without applying corrections for multiple comparisons (Table 2). The annual increase in mFF was only modestly related to the baseline severity of fatty degeneration (*r* = −0.35, *p* < 0.0001; Figure S4).

**TABLE 2:** Quantitative MRI–muscular fat fraction.

	Baseline median (average) [min‐max]	Follow up median (average) [min‐max]	annual difference median (average) [min‐max]	*p*
A‐Quantitative MRI–mFF in %–Level 1 + 2				
ALL	62 (60) [35–84]	65 (62) [39–84]	0.31 (0.47) [0–1.07]	0.011*
Upper leg	73 (69) [48–81]	74 (71) [54–82]	0.20 (0.40) [0.03–1.32]	0.017*
Lower leg	36 (34) [8–67]	42 (38) [8–69]	0.63 (0.94) [−0.13–2.64]	0.011*
B‐Quantitative MRI–mFF in %–Level 3 (individual muscles, abbreviations see Table S1)				
D	67.8 (62.4) [41.0–84.5]	61.9 (62.3) [40.2–85.0]	0.12 (−0.27) [−4.18–1.57]	0.556
BB	54.7 (51.6) [15.4–82.8]	64.3 (56.9) [27.6–84.1]	0.31 (1.03) [−0.81–2.93]	0.071
RA	49.3 (49.5) [16.1–83.0]	47.1 (51.2) [15.8–81.8]	0.01 (0.35) [−0.4–1.55]	0.146
Gmax	44.7 (52.3) [26.8–86.3]	48.8 (55.1) [36.0–85.8]	0.25 (0.57) [−0.93–2.03]	0.071
VL	76.5 (75.0) [64.0–83.5]	75.6 (76.5) [67.7–84.4]	0.1 (0.33) [−0.26–1.39]	0.085
VI	78.8 (75.4) [61.1–82.2]	77.2 (77.0) [67.2–83.4]	0.17 (0.3) [−0.79–1.5]	0.221
VM	79.2 (72.9) [51.4–83.6]	79.4 (75.0) [61.3–83.7]	0.32 (0.34) [−1.04–1.81]	0.085
RF	71.5 (71.2) [46.1–84.0]	73.1 (72.9) [47.2–85.4]	0.28 (0.45) [−0.57–1.65]	0.052
SAR	83.7 (77.6) [58.3–84.7]	83.9 (78.5) [61.7–85.1]	0.09 (0.17) [−0.25–0.63]	0.085
GR	80.4 (70.7) [26.4–83.5]	79.2 (74.4) [49.7–83.6]	0.22 (0.89) [−0.28–5.75]	0.062
BFCB	49.8 (49.3) [27.0–70.8]	51.3 (50.7) [28.7–72.5]	0.45 (0.46) [−0.84–1.8]	0.136
BFCL	80.5 (71.8) [31.1–88.0]	80.9 (73.9) [43.0–86.1]	0.16 (0.42) [−0.41–2.19]	0.172
ST	79.8 (74.4) [51.6–84.6]	80.9 (75.8) [52.7–84.5]	0.24 (0.31) [−0.05–0.87]	0.053
SM	70.9 (63.7) [32.6–79.5]	70.1 (65.4) [39.4–82.2]	0.52 (0.4) [−0.88–1.66]	0.080
ADD	65.7 (60.8) [14.1–87.1]	71.3 (62.5) [17.3–87.1]	0.14 (0.37) [−1.67–2.77]	0.231
TA	32.5 (35.9) [7.5–75.7]	45.0 (41.4) [5.9–78.5]	0.58 (1.29) [−0.38–4.64]	0.029*
ED	19.3 (20.1) [8.8–41.8]	23.6 (21.9) [8.5–41.6]	−0.01 (0.59) [−0.23–3.06]	0.101
FL	30.2 (38.1) [10.8–81.4]	33.8 (40.7) [10.7–82.7]	0.54 (0.69) [−0.02–1.37]	0.011*
GCCL	25.6 (32.6) [6.3–74.8]	32.8 (38.3) [5.8–75.4]	0.97 (1.19) [−1.04–3.24]	0.030*
GCCM	39.8 (42.7) [7.6–75.9]	43.5 (45.9) [8.1–78.1]	0.68 (0.62) [−0.82–2.16]	0.031*

*Note:* Quantitative MRI–muscular fat fraction in % (mFF) at Baseline, Follow‐Up and annual change. Data were analyzed using a three‐step hierarchical approach, subsequent levels were only analyzed if the preceding level showed statistically significant. First level = average mean mFF of all muscles (ALL). Second level = averaged upper leg and lower leg mFF. The third level = individual muscles. *p*‐Values for one sided Wilcoxon‐Test comparing BL and FU. *statistically significant at *p* < 0.05.

Notably, in contrast to the qMRI findings, no consistent changes were observed in the annual change rates of clinical measures of disease severity (Figure 2D, Table 1B), including 6MWT (average absolute change −18.5 m, range: −119–42 m; average annual change −2.87 m, range: −21.9–10.4 m; *p* = 0.37), HFMS‐EXP (average absolute change −0.22, range: −7–5; average annual change 0.11, range: −1.29–2.35; *p* = 0.47), RULM (average absolute change 1.67, range: −7–3; average annual change −0.20, range: −1.29–1.41; *p* = 0.10), and CK levels (average absolute change −2.92 μkat/l, range: −10.16–1.8 μkat/l; average annual change −0.66 μkat/l, range: −2.51–0.38 μkat/l; *p* = 0.03). Consequently, no relevant correlations were identified between annual changes in mFF and clinical outcomes (Figure 2E; Table S3).

## 4 Discussion

There is an imminent need for reliable biomarkers to monitor disease progression and therapeutic response in adults with slowly progressing forms of 5q‐SMA. This pilot study therefore evaluated quantitative MRI (qMRI) measures for long‐term monitoring in this patient population. The data presented here represent the longest observational period (median 54 months; range: 25–65 months) of qMRI assessment in SMA patients reported to date.

Notably, neither muscular fat fraction (mFF) nor clinical measures of disease severity at baseline showed a meaningful association with the age at symptom onset or the age at MRI. This likely reflects the pronounced phenotypic variability in late‐onset 5q‐SMA [28]. However, there was a good to excellent correlation between mFF and clinical measures of disease severity. This relationship was most robust when considering the average fat fraction across all studied muscles, all muscles of the upper or the lower leg, respectively. Importantly, even at the level of single muscles, significant correlations were observed between mFF and clinical severity, particularly in posterior thigh and adductor muscles.

Interestingly, the Revised Upper Limb Module (RULM) score, which specifically assesses upper extremity function in SMA, correlated only with the fat fraction of upper extremity, shoulder, and trunk muscles. However, as most individuals in this study were ambulatory (7/9) and achieved high or near‐maximal RULM scores, a ceiling effect may have influenced the correlation analysis. In contrast, the Hammersmith Functional Motor Scale Expanded (HFMS+EXP) and the 6‐Minute Walk Test (6MWT)–both of which evaluate lower extremity function–showed significant correlations with the averaged muscular fat fraction of the upper and lower legs, as well as with individual leg muscle fat fractions.

Of note, fat fractions of the hamstrings and adductor magnus showed the strongest correlations with clinical performance measures. However, all quadriceps muscles showed advanced fatty replacement, as previously reported [29]. This extensive degeneration may render these muscles less capable of capturing interindividual differences in disease severity. In contrast, muscles such as the hamstrings and adductors, which may be affected at a less advanced stage, could provide more informative biomarkers for clinical function.

Regarding longitudinal dynamics, a consistent increase in muscular fat fraction was observed over the median observational period of 5.4 years. Although this trend was evident across nearly all individual muscles studied, statistical significance was only achieved when analyzing the averaged fat fraction of all muscles, as well as pooled measures of the upper and lower legs. This likely reflects both the small sample size and the increased robustness of averaged measures. Therefore, aggregating data from a larger set of muscles may offer a more robust approach for detecting disease progression compared to assessments of individual muscles. However, the manual segmentation required to analyze a broad set of muscles is time‐consuming and may not be feasible for routine clinical application. The implementation of automated, AI‐assisted segmentation techniques could substantially enhance the feasibility of qMRI in routine clinical workflows [30, 31].

Our findings, which indicate a progressive increase in muscular fat fraction over time, are consistent with a recent longitudinal study by Otto et al. [26]. However, they contrast with recent reports by Gallone et al. and Sprenger‐Svačina et al., both of whom did not observe significant longitudinal changes. These discrepancies likely relate to shorter observational periods and the focus on individual muscles rather than composite measures [24, 25].

Quantifying the precise impact of Nusinersen treatment on the observed longitudinal changes remains challenging within the scope of this study. A prior investigation by Otto et al. reported a mean annual increase in muscular fat fraction of 1.28% in an untreated cohort of SMA patients, whereas our cohort exhibited a comparatively lower annual increase of 0.47% [26]. However, substantial differences between the cohorts preclude a direct comparison. As such, it remains speculative whether the smaller magnitude of change observed in our cohort reflects a therapeutic effect of Nusinersen. Study designs allowing baseline slope estimation prior to treatment initiation would be informative but raise ethical concerns in the context of an available effective therapy.

In contrast to the qMRI‐based measures applied in this study, traditional clinical parameters did not show significant changes over the observational period. This aligns with the slower disease progression typically observed in adult‐onset cases. These results highlight the limited sensitivity of conventional clinical scores for detecting subtle longitudinal changes at the individual level. Consequently, qMRI parameters may represent a more sensitive complementary tool for longitudinal monitoring.

This study has several limitations that should be considered. First, the small sample size limits statistical power and generalizability. Second, the absence of a non‐disease control group represents an additional limitation. Nevertheless, previous studies have consistently shown significant differences in muscular fat content between SMA patients and healthy individuals [21, 24, 32], which aligns with our own experience from an ongoing study in healthy volunteers. Although age‐related changes in muscle fat content cannot be entirely excluded, prior longitudinal and cross‐sectional studies suggest only minimal increases over time in healthy individuals [33, 34, 35, 36]. Based on this evidence, we believe the changes observed in our study predominantly reflect SMA progression rather than normal muscle aging. Finally, imaging parameters were quantified manually, introducing potential inter‐rater variability. However, all measurements were performed by a blinded investigator. Future studies should consider automated or semi‐automated approaches to improve reproducibility and efficiency.

In conclusion, this study provides evidence that qMRI represents a sensitive and objective tool for monitoring disease progression in adults with SMA, potentially surpassing conventional clinical measures in detecting subtle changes. These findings support the inclusion of qMRI‐based parameters as valuable biomarkers in the clinical assessment of SMA. However, further studies involving larger cohorts are needed to confirm these results and ensure their generalizability.

## Author Contributions

**Benjamin Troppa:** methodology, data curation, investigation, writing – review and editing, resources. **Caroline Deborah Stapf:** investigation, writing – review and editing. **Max Obenauf:** investigation, writing – review and editing. **Ilka Schneider:** investigation, writing – review and editing. **Alexander Mensch:** conceptualization, methodology, data curation, investigation, visualization, writing – original draft, supervision, formal analysis, validation. **Anna Katharina Koelsch:** investigation, writing – review and editing. **David Strube:** investigation, writing – review and editing. **Karl‐Stefan Delank:** resources, writing – review and editing. **Sebastian Plutz:** investigation, writing – review and editing. **Thomas Kendzierski:** investigation, writing – review and editing. **Torsten Kraya:** investigation, writing – review and editing. **Markus Otto:** methodology, investigation, writing – review and editing. **Dietrich Stoevesandt:** methodology, data curation, investigation, writing – review and editing, resources. **Steffen Naegel:** conceptualization, methodology, data curation, investigation, visualization, writing – original draft, supervision, formal analysis, validation.

## Conflicts of Interest

The authors declare no conflicts of interest.

## Supporting information


**Figure S1:** Baseline relationships between muscle fat fraction (mFF) of the individual muscles and disease history.
**Figure S2:** Relationships between quantitative MRI parameters averaged across upper and lower legs or individual muscles and markers of clinical severity.
**Figure S3:** Longitudinal dynamics of quantitative MRI (qMRI) parameters at the level of individual muscles.
**Figure S4:** Relationship between mFF of the individual muscle at baseline (all patients) and annual change of mFF.
**Table S1:** Analysed muscles, regions, and used abbreviations.
**Table S2A:** Baseline relationship (Spearman's Correlation) between mFF (level I + II) and clinical parameter.
**Table S2B:** Baseline relationship (Spearman's Correlation) between mFF (level III) and clinical parameter.
**Table S3:** Correlation of the differences (baseline–follow‐up).

## Data Availability

The data that support the findings of this study are available from the corresponding author upon reasonable request.
