# Supplementary material for: Quantitative Whole‐Body Muscle MRI in Adults With Spinal Muscular Atrophy–A Sensitive Tool for Long‐Time Evaluation of Disease Progression
Source: Eur J Neurol. 2026 Mar 23;33(3):e70579. doi: 10.1111/ene.70579 (PMC13093307; doi:10.1111/ene.70579)
Supplement: Supplementary file 1 — Figure S1: Baseline relationships between muscle fat fraction (mFF) of the individual muscles and disease history. Figure S2: Relationships between quantitative MRI parameters averaged across upper and lower legs or individual muscles and markers of clinical severity. Figure S3: Longitudinal dynamics of quantitative MRI (qMRI) parameters at the level of individual muscles. Figure S4: Relationship between mFF of the individual muscle at baseline (all patients) and annual change of mFF. Table S1: Analysed muscles, regions, and used abbreviations. Table S2A: Baseline relationship (Spearman's Correlation) between mFF (level I + II) and clinical parameter. Table S2B: Baseline relationship (Spearman's Correlation) between mFF (level III) and clinical parameter. Table S3: Correlation of the differences (baseline–follow‐up). [file ENE-33-e70579-s001.pdf]

Electronic Supplementary material

Article name:

Quantitative whole-body muscle MRI in adults with Spinal Muscular Atrophy - a sensitive tool for evaluation of disease progression

Authors:

Alexander Mensch, Benjamin Troppa, Ilka Schneider, Caroline Deborah Stapf, Anna Katharina Koelsch, Thomas Kendzierski, David Strube, Sebastian Plutz, Max Obenauf, Karl-Stefan Delank, Torsten Kraya, Markus Otto, Dietrich Stoevesandt, Steffen Naegel

Submitting and corresponding author:

Alexander Mensch, MD
Department of Neurology
University Medicine Halle
Ernst-Grube-Str. 40
06120 Halle (Saale), Germany
Tel.: +49 345 557 2856
Fax: +49 345 557 2860
E-Mail: alexander.mensch@medizin.uni-halle.de

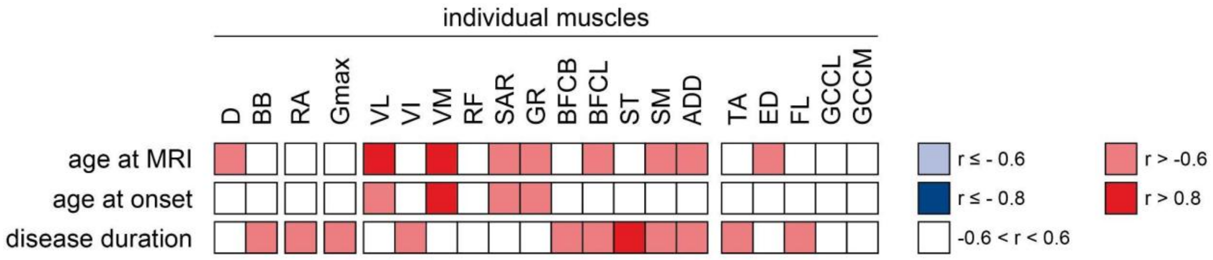

Supplementary Figure 1: Baseline relationships between muscle fat fraction (mFF) of the individual muscles and disease history.

Correlation matrix showing baseline associations between key disease history variables (age at MRI, age at disease onset, disease duration) and average mFF in the individual muscles.

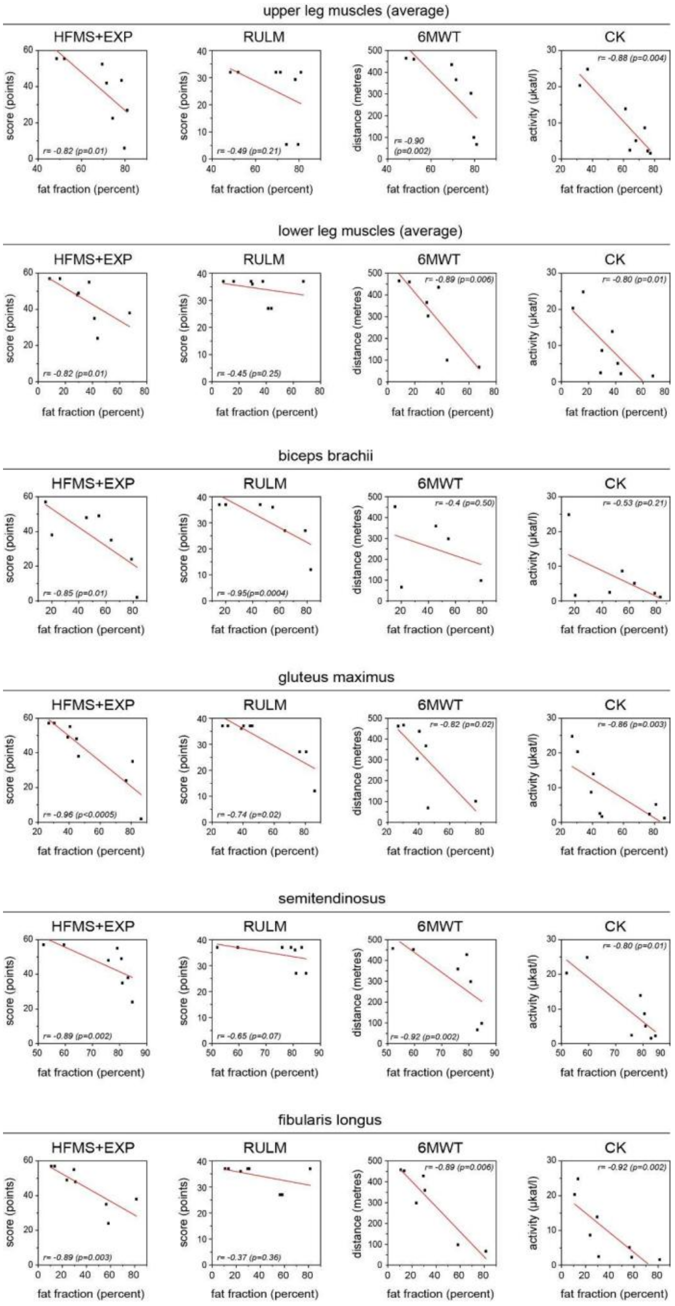

Supplementary Figure 2: Relationships between quantitative MRI parameters averaged across upper and lower legs or individual muscles and markers of clinical severity

Representative scatterplots illustrating the correlations between the averaged mFF of upper legs (UL), lower legs (LL) or of selected individual muscles in all studied body regions and clinical parameters: Hammersmith Functional Motor Scale–Expanded (HFMS+EXP), Revised Upper Limb Module (RULM), 6-Minute Walk Test (6MWT), and serum creatine kinase levels (CK).

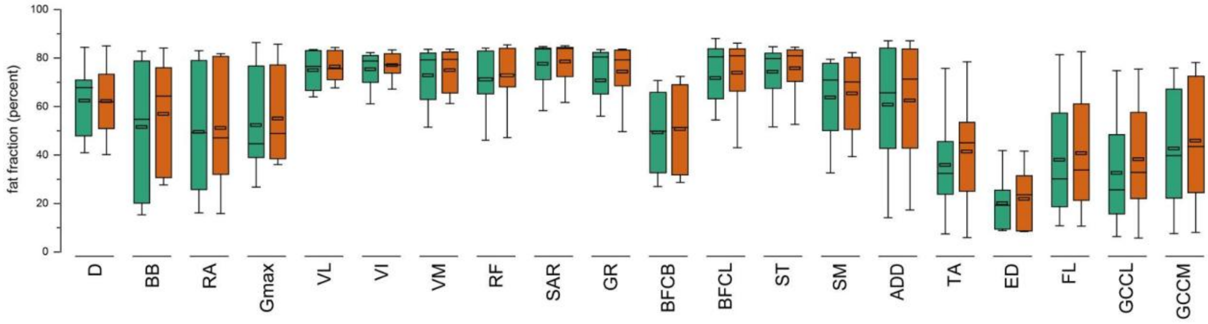

Supplementary Figure 3: Longitudinal dynamics of quantitative MRI (qMRI) parameters at the level of individual muscles

Absolute changes in muscle fat fraction (mFF) at the level of individual muscles over the observational period box-and-whisker plots summarize group-level data at baseline (green) and last follow-up (orange).

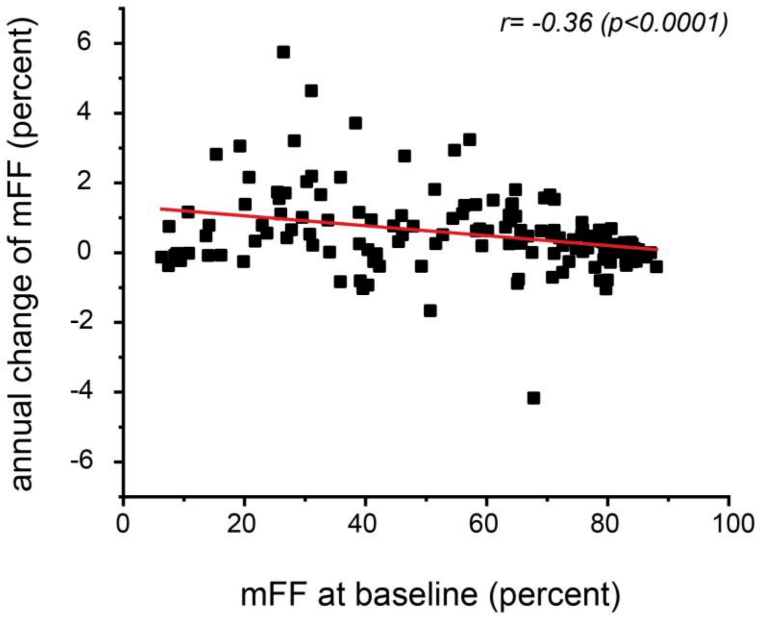

Supplementary Figure 4: Relationship between mFF of the individual muscle at baseline (all patients) and annual change of mFF

Supplementary Table S1: Analysed muscles, regions, and used abbreviations

Region	Muscle	Abbreviation
Arm*	deltoid muscle	D
	biceps brachii muscle	BB
Trunk*	rectus abdominis muscle	RA
	gluteus maximus muscle	Gmax
Upper Leg (UL)	vastus lateralis muscle	VL
	vastus intermedius muscle	VI
	vastus medialis muscle	VM
	rectus femoris muscle	RF
	sartorius muscle	SAR
	gracilis muscle	GR
	biceps femoris caput breve	BFCB
	biceps femoris caput longum	BFCL
	semitendinosus muscle	ST
	semimembranosus muscle	SM
	adductor magnus muscle	ADD
Lower leg (LL)	tibialis anterior muscle	TA
	extensor digitorum muscle	ED
	flexor digitorum longus muscle	FL

	gastrocnemius caput laterale	GCCL
	gastrocnemius caput mediale	GCCM

*Not regionwise analysed as these groups consisted of only two muscles

Supplementary Table S2-A: Baseline relationship (Spearman's Correlation) between mFF (level I+II) and clinical parameter

		ALL	UL	LL	HFMS	HFMS+	RULM	6MWT	CK
UL	Spearman's rho	0.946***							
	p-value	< .001							
	Fisher's z	1.793							
LL	Spearman's rho	0.970***	0.881**						
	p-value	< .001	0.007						
	Fisher's z	2.094	1.380						
HFMS	Spearman's rho	-0.962***	-0.868**	-0.880**					
	p-value	< .001	0.005	0.004					
	Fisher's z	-1.973	-1.323	-1.374					
HFMS+	Spearman's rho	-0.916***	-0.826*	-0.826*	0.970***				
	p-value	< .001	0.011	0.011	< .001				
	Fisher's z	-1.563	-1.177	-1.177	2.100				
RULM	Spearman's rho	-0.732*	-0.495	-0.454	0.860**	0.802**			
	p-value	0.025	0.212	0.259	0.003	0.009			
	Fisher's z	-0.934	-0.542	-0.489	1.295	1.103			
6MWT	Spearman's rho	-0.955***	-1.000***	-0.893*	0.946**	0.919**	0.490		
	p-value	< .001	< .001	0.012	0.001	0.003	0.264		
	Fisher's z	-1.886	-18.368	-1.436	1.789	1.582	0.536		
CK	Spearman's rho	-0.879**	-0.881**	-0.810*	0.857**	0.912***	0.550	0.929**	
	p-value	0.002	0.007	0.022	0.003	< .001	0.125	0.007	
	Fisher's z	-1.370	-1.380	-1.126	1.283	1.540	0.619	1.648	
Disease Duration	Spearman's rho	0.903***	0.783*	0.795*	-0.881**	-0.781*	-0.763*	-0.685	-0.723*
	p-value	< .001	0.022	0.018	0.002	0.013	0.017	0.090	0.028
	Fisher's z	1.488	1.054	1.086	-1.382	-1.047	-1.004	-0.838	-0.913

Abbreviation: ALL = averaged muscular fat fraction (mFF) of all muscles, UL = averaged mFF of the upper leg; LL = averaged mFF of the lower leg; HFMS = Hammersmith Functional Motor Scale, HFMS+ = HFMS - Expanded , RULM = Revised upper limb module for spinal muscular atrophy, 6MWT = 6 minute walking test , CK = creatine kinase

* p < .05, ** p < .01, *** p < .001

Supplementary Table S2-B: Baseline relationship (Spearman's Correlation) between mFF (level III) and clinical parameter

		HFMS	HFMS+	RULM	6MWT	CK
D	Spearman's rho	-0.698*	-0.736*	-0.743*	-0.357	-0.633
	p-value	0.037	0.024	0.022	0.444	0.076
BB	Spearman's rho	-0.893*	-0.857*	-0.954***	-0.400	-0.536
	p-value	0.012	0.024	< .001	0.517	0.236
RA	Spearman's rho	-0.918**	-0.973***	-0.666	-0.841*	-0.901**
	p-value	0.004	< .001	0.102	0.036	0.006
Gmax	Spearman's rho	-0.924***	-0.962***	-0.743*	-0.821*	-0.867**
	p-value	< .001	< .001	0.022	0.034	0.005
VL	Spearman's rho	-0.472	-0.506	-0.085	-0.805*	-0.749*
	p-value	0.238	0.200	0.841	0.029	0.033
VI	Spearman's rho	-0.780*	-0.655	-0.501	-0.901**	-0.675
	p-value	0.022	0.078	0.206	0.006	0.066
VM	Spearman's rho	-0.542	-0.575	-0.536	-0.571	-0.571
	p-value	0.165	0.136	0.171	0.200	0.151
RF	Spearman's rho	-0.079	-0.145	0.373	-0.703	-0.527
	p-value	0.853	0.733	0.362	0.078	0.180
SAR	Spearman's rho	-0.821*	-0.854**	-0.686	-0.837*	-0.741*
	p-value	0.013	0.007	0.060	0.019	0.036
GR	Spearman's rho	-0.585	-0.691	-0.188	-0.764*	-0.819*
	p-value	0.127	0.058	0.656	0.046	0.013
BFCB	Spearman's rho	-0.807*	-0.838**	-0.412	-0.714	-0.762*
	p-value	0.015	0.009	0.310	0.088	0.037
BFCL	Spearman's rho	-0.715*	-0.566	-0.477	-0.811*	-0.611
	p-value	0.046	0.143	0.232	0.027	0.108
ST	Spearman's rho	-0.945***	-0.867**	-0.643	-0.929**	-0.802*
	p-value	< .001	0.005	0.085	0.007	0.017
SM	Spearman's rho	-0.764*	-0.627	-0.539	-0.857*	-0.623
	p-value	0.027	0.096	0.168	0.024	0.099
ADD	Spearman's rho	-0.723*	-0.647	-0.536	-0.857*	-0.667
	p-value	0.043	0.083	0.171	0.024	0.083
TA	Spearman's rho	-0.711*	-0.695	-0.454	-0.786*	-0.619
	p-value	0.048	0.056	0.259	0.048	0.115
ED	Spearman's rho	-0.679	-0.736	-0.319	-0.899*	-0.883**
	p-value	0.094	0.059	0.485	0.015	0.008
FL	Spearman's rho	-0.843**	-0.898**	-0.371	-0.893*	-0.929**
	p-value	0.009	0.002	0.365	0.012	0.002
GCCL	Spearman's rho	-0.482	-0.419	-0.041	-0.679	-0.524
	p-value	0.227	0.301	0.923	0.110	0.197
GCCM	Spearman's rho	-0.667	-0.687	-0.249	-0.667	-0.707
	p-value	0.071	0.060	0.552	0.102	0.050

Abbreviation: D to GCCM = muscular fat fraction (mFF) of individual muscles, abbreviations see table S1; HFMS = Hammersmith Functional Motor Scale, HFMS+ = HFMS - Expanded , RULM = Revised upper limb module for spinal muscular atrophy, 6MWT = 6 minute walking test , CK = creatine kinase
* p < .05, ** p < .01, *** p < .001

Supplementary Table S3: Correlation of the differences (baseline - follow-up)

		Δ ALL	Δ UL	Δ LL	Δ HFMS+	Δ RULM	Δ 6MWT
**Δ UL**	Spearman's rho	**0.762***					
	p-value	0.037					
	Fisher's z	1.001					
**Δ LL**	Spearman's rho	0.357	-0.190				
	p-value	0.389	0.665				
	Fisher's z	0.374	-0.193				
**Δ HFMS+**	Spearman's rho	-0.152	0.422	-0.386			
	p-value	0.696	0.298	0.346			
	Fisher's z	-0.153	0.450	-0.407			
**Δ RULM**	Spearman's rho	-0.256	-0.037	-0.173	**0.727***		
	p-value	0.505	0.931	0.682	0.026		
	Fisher's z	-0.262	-0.037	-0.175	0.923		
**Δ 6MWT**	Spearman's rho	0.029	0.257	-0.086	0.522	0.794	
	p-value	1.000	0.658	0.919	0.288	0.059	
	Fisher's z	0.029	0.263	-0.086	0.579	1.083	
**Δ CK**	Spearman's rho	-0.083	-0.381	0.452	0.110	-0.051	-0.543
	p-value	0.843	0.360	0.267	0.779	0.896	0.297
	Fisher's z	-0.084	-0.401	0.488	0.110	-0.051	-0.608

Abbreviation: Δ = difference between baseline and follow up for: ALL = averaged muscular fat fraction (mFF) of all muscles, UL = averaged mFF of the upper leg; LL = averaged mFF of the lower leg; HFMS = Hammersmith Functional Motor Scale, HFMS+ = HFMS - Expanded , RULM = Revised upper limb module for spinal muscular atrophy, 6MWT = 6 minute walking test , CK = creatine kinase
* $p < .05$
